# Kleine-levin syndrome

**DOI:** 10.1192/j.eurpsy.2021.667

**Published:** 2021-08-13

**Authors:** R. Mota Freitas, M.T. Valadas

**Affiliations:** 1 Departamento De Psiquiatria E Saúde Mental, Hospital do Espírito Santo de Évora, Évora, Portugal; 2 Serviço De Psiquiatria, Unidade Local de Saúde do Baixo Alentejo, Beja, Portugal

**Keywords:** Kleine-Levin Syndromept

## Abstract

**Introduction:**

Kleine-Levin Syndrome (KLS) is an extremely rare disorder of unknown etiology. It affects mainly male adolescents and is thought to follow a relapse-remitting course. During episodes of illness, a wide array of neuropsychiatric symptoms may present and a psychiatric diagnosis might be incorrectly made.

**Objectives:**

We aim to review the literature on the clinical manifestations of KLS, as well as the current evidence regarding this disorder’s management.

**Methods:**

We performed an updated review in the PubMed database using the terms “Kleine-Levin Syndrome”. The included articles were selected by title and abstract.

**Results:**

KLS usually presents with recurrent episodes, lasting days to weeks, of severe hypersomnia, cognitive impairment, major apathy and derealization, among other neuropsychiatric symptoms. Although it was previously thought that complete normalization occurred between episodes, recent evidence suggests that around one third of patients have mild cognitive impairment and there are alterations in brain blood flow during the asymptomatic periods. During episodes of illness, management comprises environmental measures as well as drug therapy. Corticosteroids and amantadine have been successful in stopping episodes and lithium may be useful in a preventative role, however, there are no randomized controlled trials focusing on KLS treatment.

**Conclusions:**

KLS remains an elusive entity since it is an extremely rare disorder with unclear etiology, course, and no consensual treatment. Further research is warranted in this area, namely randomized controlled trials. It is important for the practicing psychiatrist to be aware of this illness in order to recognize it and adequately manage it.

